# Variability in time to surgery for patients with acute thoracolumbar spinal cord injuries

**DOI:** 10.1038/s41598-021-92310-z

**Published:** 2021-06-25

**Authors:** Jetan H. Badhiwala, Gerald Lebovic, Michael Balas, Leodante da Costa, Avery B. Nathens, Michael G. Fehlings, Jefferson R. Wilson, Christopher D. Witiw

**Affiliations:** 1 Division of Neurosurgery, Department of Surgery, University of Toronto, Toronto, ON Canada; 2 Li Ka Shing Knowledge Institute, St. Michael’s Hospital, 30 Bond Street, TorontoToronto, ON M5W 1W8 Canada; 3 Institute of Health Policy Management and Evaluation, University of Toronto, Toronto, ON Canada; 4 Spine Program, Department of Surgery, University of Toronto, Toronto, ON Canada; 5 Sunnybrook Research Institute, Sunnybrook Healthsciences Center, 2075 Bayview Avenue, Toronto, ON M4N 3M5 Canada; 6 Division of General Surgery, Department of Surgery, University of Toronto, Toronto, ON Canada; 7 Medical Director, Trauma Quality Improvement Program, American College of Surgeons, Chicago, IL USA; 8 Spine Program, Krembil Brain Institute, Toronto Western Hospital, 399 Bathurst St, Toronto, ON M5T 2S8 Canada

**Keywords:** Trauma, Spinal cord diseases, Epidemiology

## Abstract

There are limited data pertaining to current practices in timing of surgical decompression for acute thoracolumbar spinal cord injury (SCI). We conducted a retrospective cohort study to evaluate variability in timing between- and within-trauma centers in North America; and to identify patient- and hospital-level factors associated with treatment delay. Adults with acute thoracolumbar SCI who underwent decompressive surgery within five days of injury at participating trauma centers in the American College of Surgeons Trauma Quality Improvement Program were included. Mixed-effects regression with a random intercept for trauma center was used to model the outcome of time to surgical decompression and assess risk-adjusted variability in surgery timeliness across centers. 3,948 patients admitted to 214 TQIP centers were eligible. 28 centers were outliers, with a significantly shorter or longer time to surgery than average. Case-mix and hospital characteristics explained < 1% of between-hospital variability in surgical timing. Moreover, only 7% of surgical timing variability within-centers was explained by case-mix characteristics. The adjusted intraclass correlation coefficient of 12% suggested poor correlation of surgical timing for patients with similar characteristics treated at the same center. These findings support the need for further research into the optimal timing of surgical intervention for thoracolumbar SCI.

## Introduction

Acute spinal cord injury (SCI) is a devastating condition that exerts a significant physical, mental, and economic burden on patients, caregivers, and society at large^1,2^. While there is robust clinical evidence to support superior neurological outcomes with early (< 24 h) compared to late (≥ 24 h) decompression for cervical SCI, the efficacy of early decompressive surgery in the setting of thoracolumbar SCI remains a critical knowledge gap^3–6^. Thoracolumbar injuries have distinct biomechanical and physiological characteristics; most notably, these injuries often result from higher energy mechanisms, and the tenuous vascular supply to the spinal cord in this region may lead to less potential for recovery^7,8^. The natural history of complete thoracolumbar SCI has been thought to involve negligible recovery, which has made it the target of novel experimental therapeutics^8–11^.

In addition to the relative equipoise in timing of decompression for patients with acute thoracolumbar SCI, there can be several barriers to urgent spinal surgical intervention in this population. At the patient-level, individuals with a thoracolumbar SCI often have concomitant chest, abdominal, pelvic, and/or extremity injuries, which may themselves necessitate intervention^12,13^. Further, the hemodynamic status of the patient may be prohibitive^14,15^. At the hospital level, physician decision-making, institutional policies, and resource allocation are likely to influence intervention^16^.

Overall, there is little agreement on what constitutes an optimal threshold for surgical timing for a patient with a thoracolumbar SCI. Thresholds used in previous studies range from 8 to 72 h^17–23^. There is hence likely to be substantial variability, both between and within trauma centers, with regard to timing of surgical decompression. To that end, in patients with thoracolumbar SCI, this study aimed to (1) identify patient- and hospital-level factors independently associated with timing of surgical decompression; and (2) estimate variability in time to decompressive surgery between and within trauma centers. Rather than a survey of physician preferences, this study provides a snapshot of actual current practice at trauma centers across North America.

## Materials and methods

### Data source

Data were derived from the American College of Surgeons (ACS) Trauma Quality Improvement Program (TQIP) database for 2010 to 2016^24,25^. TQIP collects data from more than 450 ACS- and state-verified level I and II trauma centers across the United States and Canada. Patients with at least one severe injury (Abbreviated Injury Scale [AIS] ≥ 3 in at least one body region) are included. Quality and reliability of the data are ensured through rigorous training of data abstractors and inter-rater reliability audits of participating sites.

### Subjects

Adult patients (≥ 16 years) who underwent surgical decompression for acute thoracolumbar SCI due to blunt trauma were eligible. Patients with a diagnosis of acute cervical or thoracolumbar SCI were first identified using Abbreviated Injury Scale (AIS) codes (Supplementary Table 1). Surgical procedures were identified using International Classification of Diseases, 9th/10th revision, Procedure Classification System (ICD-9-PCS and ICD-10-PCS) codes specifying spinal decompression and/or fusion (Supplementary Table 2). Patients were excluded if: (1) they had a non-survivable injury in any body region (AIS 6); (2) they had a cervical SCI; (3) they suffered penetrating trauma; (4) they did not undergo spinal surgical decompression; or (5) they underwent spinal surgical intervention more than 5 days after injury. The threshold of 5 days corresponds to the 90th percentile for time to surgery and was selected because prolonged wait times beyond this may be attributable to non-modifiable patient factors rather than modifiable system/quality factors. Further, patients treated at trauma centers with fewer than 5 patients meeting the above criteria over the study period were excluded. This study was approved by the Sunnybrook Health Sciences Center research ethics board (Toronto, Ontario, Canada), and all methods were carried out in accordance with relevant guidelines and regulations. This study used only de-identified retrospective patient data, and individual participant informed consent was waived by the research ethics board. All methods were carried out in accordance with STROBE guidelines and regulations.

### Patient-and Hospital-level characteristics

Patient demographic characteristics included age, sex, race, insurance type, and comorbidities. Comorbid status was evaluated by a modified Charlson Comorbidity Index (mCCI) (Supplementary Table 3). Injury characteristics included mechanism of injury, completeness of neurological injury, AIS score from each body region, total Injury Severity Score (ISS), and year of injury. Presenting emergency department (ED) characteristics included need for assisted ventilation, hypotension (systolic blood pressure [SBP] < 90 mmHg), Glasgow Coma Scale (GCS) score, positive test for alcohol or other drug, and whether the patient was transferred from another institution. Hospital characteristics included geographic region, trauma center level, number of beds, teaching status, and volume (i.e., number of cases treated per year that fulfilled eligibility criteria for inclusion in the present study).

### Outcomes

The primary outcome was time to decompression, defined as hours elapsed from ED arrival to first spinal surgical intervention.

### Statistical analysis

All statistical analyses were performed using R version 3.5.0 (R Foundation for Statistical Computing, Vienna, Austria) with an a priori specified significance level of *P* = 0.05 (two-tailed). Descriptive statistics were by mean and standard deviation (SD) or median and interquartile range (IQR) for continuous variables and count and percentage for categorical variables.

A hierarchical multi-level mixed-effects regression model was constructed with time to surgical decompression as the outcome. Fixed-effect covariates were specified for patient-level demographic, injury, and presenting ED characteristics, including age, sex, race, insurance type, comorbidities, mechanism of injury, AIS score from each body region, ISS, GCS, need for assisted respiration, hypotension, transfer status, positive alcohol test, positive drug test, year of injury, as well as hospital-level variables, including trauma center level, number of beds, teaching status, and volume (number of cases treated per year). A random-effect term was specified to account for clustering of patients within individual trauma centers. Missing data were not imputed and modelling was performed by ‘complete case analysis’.

#### Factors associated with time to surgical decompression

The fixed-effects output of the hierarchical regression model was used to test the independent association of baseline factors with time to surgical decompression, adjusting for patient- and hospital-level confounders. Effect sizes were summarized by mean differences (MDs), and associated bootstrapped 95% confidence intervals (CIs), as residuals violated the normaility assumption.

#### Variability in practice patterns of time to surgical decompression

Variability across trauma centers in the timing of decompression, adjusting for individual case-mix and hospital characteristics, was evaluated using the random-effects output of the multi-level regression model. Each hospital’s unique risk-adjusted MD and associated 95% CI for time to surgical decompression was derived from the model, and outliers were identified. A ‘low outlier’ (i.e., significantly shorter time to decompressive surgery than average) was a hospital where the upper limit of the 95% CI fell below 0. By contrast, a ‘high outlier’ (i.e., significantly longer time to decompressive surgery than average) was a hospital where the lower limit of the 95% CI was greater than 0.

Next we determined the extent that variability in surgical timing resulted from differences in hospital case-mix and characteristics. To this end, we compared the aforementioned ‘full model’, which included fixed-effect covariates for case-mix and hospital characteristics with a ‘null model’ that contained no explanatory variables. The difference in hospital-level variance between the ‘full model’ (V_H full_) and the ‘null model’ (V_H null_) represents the variability between hospitals in timeliness of surgery that is attributable to the case-mix and hospital characteristics^26^. This proportional change in variance (PCV) at the hospital level is reported as the hospital PCV (PCV_H_) (Eq. 1).

1 $$PCV_{H} = \frac{{V_{{H~null}} - ~V_{{H~full}} }}{{V_{{H~null}} }}$$

The effect of individual characteristics on within-hospital variability in surgical timing was determined by calculating the difference in individual-level variance between the ‘full model’ (V_I full_) and the ‘null model’ (V_I null_)^26^. This PCV at the individual level is reported as the individual PCV (PCV_I_) (Eq. 2).

2 $$PCV_{I} = \frac{{V_{{I~null}} - ~V_{{I~full}} }}{{V_{{I~null}} }}$$

Finally, the adjusted intraclass correlation coefficient (ICC) was used to measure the degree to which time to surgical decompression was contingent upon the individual hospital^26,27^. The adjusted ICC can be interpreted as the expected correlation in time to surgical decompression between two randomly drawn patients with similar characteristics treated at the same trauma center; or in other words, the proportion of total variance in surgical timing that remains at the hospital level after taking into account the individual patient composition and characteristics of the hospital. This is calculated using the individual-level (V_I full_) and hospital-level variance (V_H full_) from the ‘full model’ (Eq. 3).

3 $$ICC_{{Adj}} = \frac{{V_{{H~full}} }}{{V_{{H~full}} + V_{{I~full}} ~}}~~$$

## Results

### Study Population

Within the TQIP database for 2010–2016, there were 4,305 patients from 372 different trauma centers with survivable injuries who underwent surgical decompression for thoracoluimbar SCI due to blunt trauma within 5 days of injury (Supplementary Fig. 1). A total of 158 centers (357 patients) that treated fewer than 5 patients meeting eligibility criteria over the study period were excluded. The final study cohort hence consisted of 3,948 patients treated at 214 individual trauma centers. Baseline characteristics are summarized in Table 1. Mean age was 39.2 years and there were 2,995 (75.9%) males. The majority of patients were injured in a motor vehicle collision (MVC) (*n* = 2,234, 56.6%) or fall (*n* = 1,323, 33.5%). Mean time to surgical decompression was 28.5 h (median 18.8 h). The cumulative percentage of patients undergoing decompression at each time point from ED arrival is plotted in Supplementary Fig. 2.

**Table 1:** Baseline characteristics of study cohort.

**Demographic characteristics**	
Age (yrs)—mean ± SD	39.2 ± 16.9
Male—*n* (%)	2,995 (75.9)
*Race—n (%)*	
White	2,954 (74.8)
Black	369 (9.3)
Asian	59 (1.5)
Other	382 (9.7)
Unknown	184 (4.7)
*Insurance—n (%)*	
Government	1,149 (29.1)
Private	2,429 (61.5)
Other	370 (9.4)
*Modified Charlson Comorbidity Index—n (%)*	
0	2,587 (66.1)
1	640 (16.3)
2–3	530 (13.5)
> 3	158 (4.0)
**Injury/Presentation characteristics**	
*Mechanism of injury—n (%)*	
MVC	2,234 (56.6)
Fall	1,323 (33.5)
Pedestrian/bicyclist	194 (4.9)
Struck by object	156 (4.0)
Other/Unknown	37 (0.9)
*Completeness of neurological injury—n (%)*	
Complete	2,406 (60.9)
Incomplete	1,542 (39.1)
*Severe injury (AIS ≥ 3)—n (%)*	
Head	682 (17.3)
Face	19 (0.5)
Neck	43 (1.1)
Thorax	2,313 (58.6)
Abdomen	320 (8.1)
Spine	3,948 (100.0)
Upper extremities	94 (2.4)
Lower extremities	354 (9.0)
Unspecified	2 (0.05)
ISS ≥ 16—*n* (%)	3,884 (99.1)
*GCS—n (%)*	
15	2,934 (75.5)
13–14	447 (11.5)
9–12	128 (3.3)
3–8	375 (9.7)
Assisted ventilation in ED—*n* (%)	399 (10.1)
Hypotension in ED (SBP < 90)—*n* (%)	313 (8.0)
Transferred from another institution—*n* (%)	1,188 (30.1)
Positive alcohol test—*n* (%)	809 (20.5)
Positive drug test—*n* (%)	926 (23.5)
*Year—n (%)*	
2010	335 (8.5)
2011	424 (10.7)
2012	534 (13.5)
2013	589 (14.9)
2014	696 (17.6)
2015	721 (18.3)
2016	649 (16.4)
**Treatment characteristics**	
Time to surgical decompression (hrs)—mean ± SD	28.5 ± 27.3
**Hospital characteristics**	
*Region—n (%)*	
Midwest	794 (22.4)
Northeast	397 (11.2)
South	1,513 (42.8)
West	835 (23.6)
Level I trauma center—n (%)	3,157 (80.0)
*No. of beds—n (%)*	
> 500	1,989 (50.4)
251–500	1,659 (42.0)
≤ 250	300 (7.6)
*Teaching status—n (%)*	
Community	1,115 (28.2)
Non-teaching	243 (6.2)
University	2,590 (65.6)
Volume (no. of cases per year)—median (IQR)	4.5 (2.6–7.3)

*MVC* Motor Vehicle Collision;* ISS* Injury Severity Score;* GCS* Glasgow Coma Scale;* ED* Emergency Department;* SBP* Systolic Blood Pressure.

### Factors associated with time to surgical decompression

**Figure 1:**
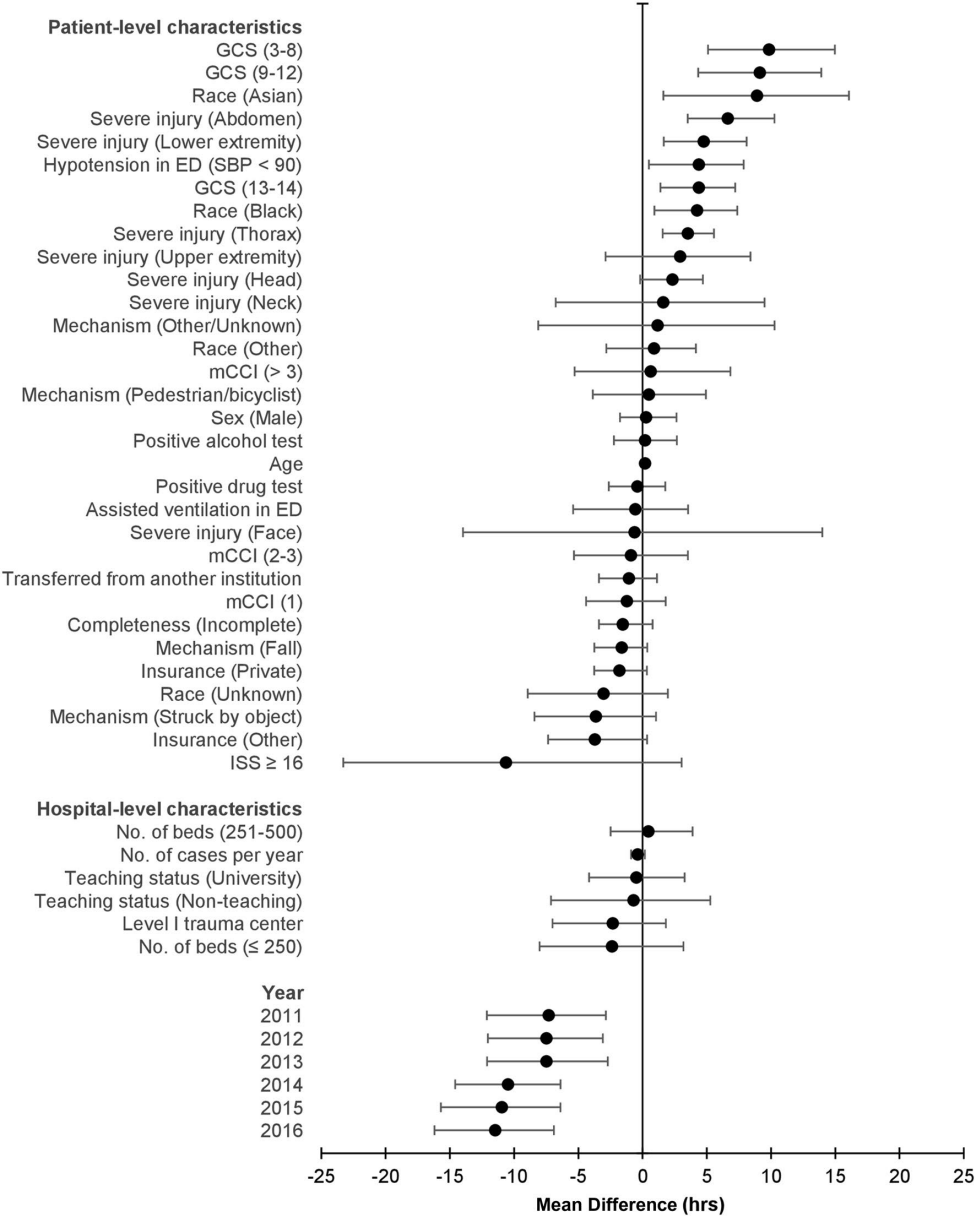
Forest plot of adjusted MDs and 95% CIs for patient- and hospital-level characteristics for time to surgical decompression^45^.

Results from the hierarchical multi-level mixed-effects regression model for time to surgical decompression are summarized in Table 2 and Fig. 1. Patient-level covariates significantly associated with longer time to decompressive surgery included older age (MD 0.18); black (MD 4.23) or Asian (MD 8.91) compared to white race; severe thorax (MD 3.51), abdomen (MD 6.63), or lower extremity (MD 4.75) injury; and hypotension (MD 4.38) or altered level of consciousness (GCS 13–14 [MD 4.38]; GCS 9–12 [MD 9.13]; GCS 3–8 [MD 9.85]) in the ED. By contrast, after 2010, each subsequent year was associated with an incrementally shorter time to surgical decompression. No center-level covariate was a significant factor in influencing time to surgical decompression.

**Table 2:** Association of patient- and hospital-level characteristics with timing of surgical decompression for acute thoracolumbar SCI.

	MD	95% CI	P Value
**Patient-level characteristics**			
Age	0.18	0.09 to 0.28	< 0.001
Male	0.27	− 1.76 to 2.64	0.427
*Race*			
White	Reference		
Black	4.23	0.92 to 7.37	0.004
Asian	8.91	1.62 to 16.06	0.004
Other	0.89	− 2.82 to 4.15	0.292
Unknown	− 3.03	− 8.94 to 1.97	0.143
*Insurance*			
Government	Reference		
Private	− 1.80	− 3.76 to 0.34	0.056
Other	− 3.71	− 7.36 to 0.35	0.054
*Modified Charlson Comorbidity Index*			
0	Reference		
1	− 1.23	− 4.40 to 1.80	0.199
2–3	− 0.90	− 5.34 to 3.53	0.324
> 3	0.64	− 5.29 to 6.84	0.427
*Mechanism*			
MVC	Reference		
Fall	− 1.61	− 3.75 to 0.37	0.060
Pedestrian/bicyclist	0.48	− 3.88 to 4.93	0.401
Struck by object	− 3.64	− 8.41 to 1.05	0.052
Other/Unknown	1.17	− 8.12 to 10.27	0.408
*Completeness of neurological injury*			
Complete	Reference		
Incomplete	− 1.55	− 3.39 to 0.78	0.058
*Severe injury (AIS* ≥ *3)*			
Head	2.32	− 0.19 to 4.69	0.053
Neck	1.60	− 6.76 to 9.49	0.351
Face	− 0.62	− 13.98 to 13.99	0466
Thorax	3.51	1.56 to 5.56	0.001
Abdomen	6.63	3.51 to 10.27	< 0.001
Upper extremities	2.92	− 2.88 to 8.40	0.156
Lower extremities	4.75	1.65 to 8.10	0.001
ISS ≥ 16	− 10.63	− 23.30 to 3.04	0.057
*GCS*			
15	Reference		
13–14	4.38	1.39 to 7.21	< 0.001
9–12	9.13	4.33 to 13.91	< 0.001
3–8	9.85	5.09 to 14.97	< 0.001
Assisted ventilation in ED	− 0.57	− 5.41 to 3.54	0.416
Hypotension in ED (SBP < 90)	4.38	0.49 to 7.87	0.005
Transferred from another institution	− 1.05	− 3.39 to 1.12	0.146
Positive alcohol test	0.19	− 2.23 to 2.67	0.438
Positive drug test	− 0.40	− 2.63 to 1.77	0.359
*Year*			
2010	Reference		
2011	− 7.31	− 12.12 to − 2.86	< 0.001
2012	− 7.48	− 12.04 to − 3.09	< 0.001
2013	− 7.49	− 12.10 to − 2.71	< 0.001
2014	− 10.47	− 14.59 to − 6.39	< 0.001
2015	− 10.95	− 15.70 to − 6.39	< 0.001
2016	− 11.47	− 16.19 to -6.90	< 0.001
**Hospital-level characteristics**			
Level I trauma center	− 2.32	− 7.01 to 1.81	0.154
*No. of beds*			
> 500	Reference		
251–500	0.45	− 2.49 to 3.89	0.389
≤ 250	− 2.38	− 8.02 to 3.18	0.214
*Teaching status*			
Community	Reference		
Non-teaching	− 0.71	− 7.13 to 5.27	0.409
University	− 0.49	− 4.16 to 3.27	0.404
No. of cases per year	− 0.39	− 0.90 to 0.18	0.091

*MVC* Motor Vehicle Collision;* ISS* Injury Severity Score;* GCS* Glasgow Coma Scale;* ED* Emergency Department;* SBP* Systolic Blood Pressure.

### Variability in practice patterns of time to surgical decompression

**Figure 2:**
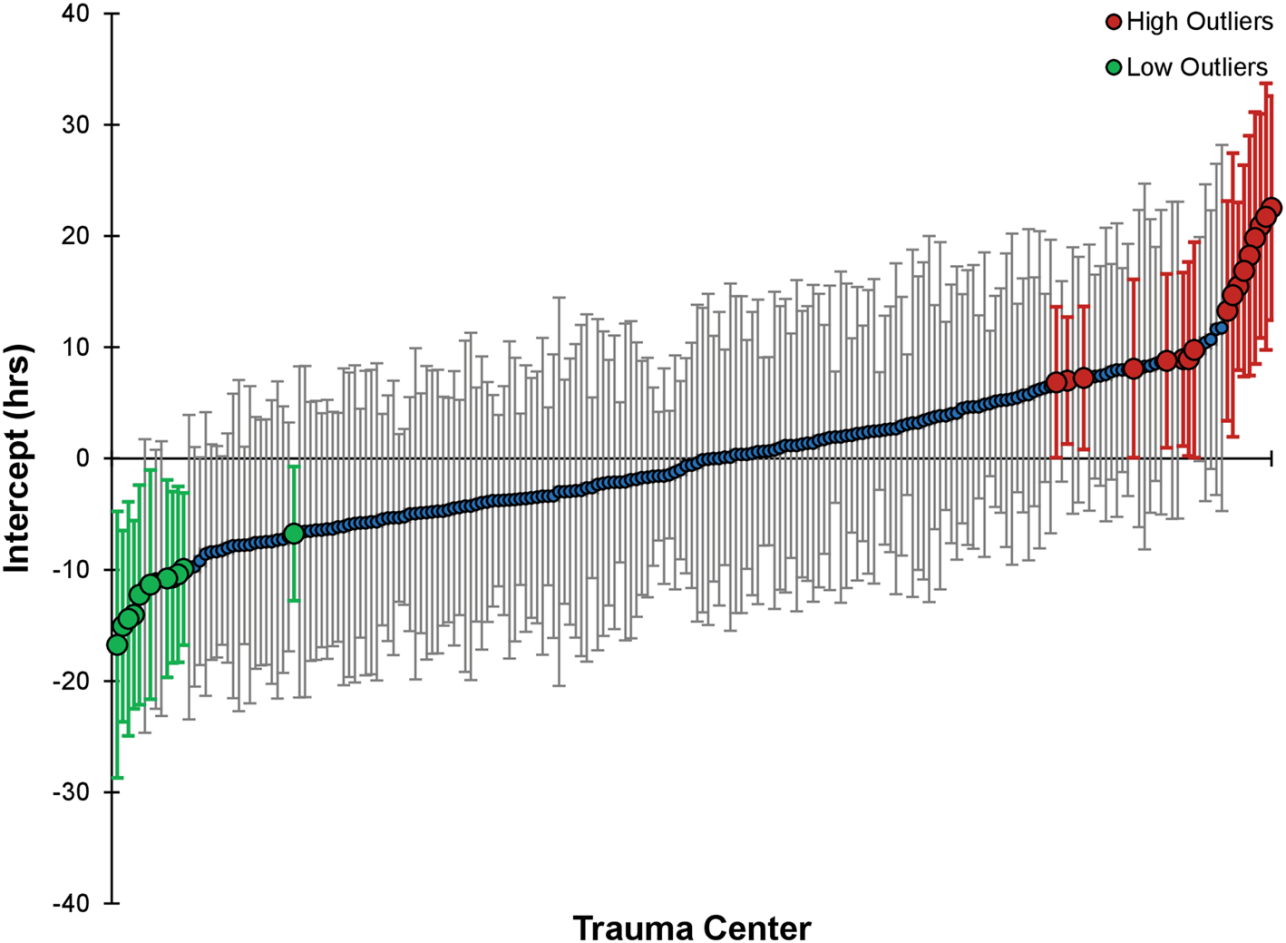
‘Caterpillar plot’ of trauma center MDs and 95% CIs for time to surgical decompression, risk-adjusted for patient- and hospital-level characteristics^45^.

Figure 2 plots each trauma center’s unique risk-adjusted mean difference for time to surgical decompression based on the random-effects output of the hierarchical multi-level mixed-effects regression model. After adjustment for case-mix and hospital-level variables, 11 hospitals were found to be low outliers, with significantly shorter time to decompressive surgery than average; by contrast, another 17 hospitals were high outliers, with significantly longer time to decompressive surgery than average. The hospital PCV between the ‘null model’ and the ‘full model’ was only 0.8%, indicating the case-mix and hospital characteristics included in the full model explained less than 1% of the variability between hospitals in time to surgery (Table 3). The individual PCV was 7.4%, suggesting individual case characteristics explained about 8% of the variability in time to surgery within patients at the same hospital. The adjusted ICC was 12.5%, indicating rather poor correlation in time to decompression between two similar patients treated at the same trauma center (Table 3). When hospitals were grouped into quintiles based on their risk-adjusted MD for time to surgical decompression, significant differences in mean time to surgery were noted. The 828 patients across the 42 hospitals in the 1st quintile had a mean time to surgery of 16.1 ± 17.9 h, while the 886 patients across the 42 hospitals in the 5th quintile had a mean surgical time of 41.8 ± 31.4 h.

**Table 3:** Estimates of variability in practice patterns in timing of decompression for acute thoracolumbar SCI.

	‘Null model’	‘Full model’
**Variance components**		
Between hospitals (V_H_)	87.4	86.7
Between individuals (V_I_)	657.5	609.1
**Proportional change in variance (PCV)**		
Between hospitals (PCV_H_)	reference	0.8%
Between individuals (PCV_I_)	reference	7.4%
Adjusted intraclass correlation (ICC_Adj_)	11.7%	12.5%

## Discussion

The rationale for early surgery after acute SCI is that the expeditious relief of mechanical compression to the spinal cord may mitigate the degree of secondary neurological injury in order to afford better long-term recovery^3,28^. There is strong basic science data derived from rodent models of SCI to indicate superior neurobehavioral outcomes when the time from injury to decompression is minimized^29–33^. The highest quality clinical study to date on this topic is the Surgical Timing in Acute Spinal Cord Injury Study (STASCIS), which found early surgery, within 24 h of injury, to be associated with a greater odds of achieving a 2-or-more grade improvement in American Spinal Injury Association (ASIA) Impairment Scale (AIS) in patients with acute cervical SCI^6^. However, the clinical data pertaining to the effect of time to surgery for acute thoracolumbar SCI are more limited, with mixed results being derived from smaller, lower quality studies. A randomized trial of 35 patients with thoracolumbar SCI demonstrated no differences in AIS grade or motor score improvement at 12 months with early (< 24 h) compared to late (24–72 h) surgical decompression; however, this trial was almost certainly underpowered to detect a difference^19^. By contrast, a registry-based study of 86 thoracolumbar SCI patients found early surgical decompression (< 24 h) to be associated with 7 points in additional motor recovery and 60% decreased incidence of pulmonary complications^8^. A recent systematic review and meta-analysis including 14 studies failed to demonstrate a significant difference in AIS grade recovery with early (< 24 h) versus late (≥ 24 h) surgical decompression^22^. Ultimately, there remains equipoise regarding the impact of time to surgical decompression in this population, which is likely to be spurring variability in practice patterns. This study sought to elucidate key factors that may be driving treatment decisions surrounding time to surgery in patients with acute thoracolumbar SCI, and moreover, to concretely quantify the variability in practice patterns between and within trauma centers in North America.

Among patient-level characteristics, older age and black or Asian race were associated with longer time to decompressive surgery. Older age has previously been associated with less aggressive treatment and longer treatment delay following acute SCI^34^. There are several possible reasons for this finding. First, there may be delay in recognizing older patients’ generally less severe injuries. Second, older patients are more likely to require some degree of pre-operative medical optimization and a more complex management scheme. However, the fact that older age was a significant predictor of longer time to surgery in this analysis which also adjusted for comorbidities suggests that there may be some element of an age-related therapeutic bias, with less perceived urgency in appropriately working-up, and developing a treatment plan for, older patients. The effect of black or Asian race highlights that socioeconomic and sociodemographic indicators appear to influence clinical decision-making in patients with acute thoracolumbar SCI. There is growing recognition of racial disparities in surgical care and outcomes. Mutiple studies have suggested that patients from visible minority groups are less likely to receive surgery^35^. Although these studies have predominantly focused on elective, non-emergent procedures, black/minority race has also been linked to greater likelihood of receiving surgical intervention for acute spinal fractures^36^. Our study, similarly, suggests race may influence clinical decision-making in the emergent setting. Surgeons should be cognizant of how sociodemographic factors may shape their own and patients’ attitudes and expectations during the clinical encounter.

With regard to injury-related characteristics, severe chest, abdominal, and lower extremity injury as well as hypotension or altered level of consciousness on arrival were associated with longer delay to decompressive surgery. These findings are perhaps intuitive and somewhat expected. The presence of concomitant injuries, particularly those that may lead to hemodynamic instability as well as traumatic brain injuries, would generally take precedence over the management of an acute SCI, as per standard Advanced Trauma Life Support (ATLS) protocol^37^.

Interestingly, incrementally shorter times to decompressive surgery were observed for each year subsequent to 2010. This likely reflects a shift in treatment paradigms for spinal cord injury and growing recognition of the role of early decompressive surgery with the emergence of more recent clinical evidence^6,20,38,39^.

In addition to identifying factors driving variability in timing of surgical decompression for acute thoracolumbar SCI, this study provides an analysis of the degree of variability in practice patterns between and within trauma centers. Twenty-eight hospitals were low or high outliers, with substantially shorter or longer time to decompressive surgery than average. Case-mix and hospital characteristics explained less than 1% of the variability in surgical timing between hospitals. This suggests that these factors are not the explaination for why certainly hospitals have substantially longer time to surgery than others. Moreover, less than 8% the variability in surgical timing within hospitals was attributable to individual patient characteristics. Hence, there remains substantial differences in surgical timing from unobserved influences, both between hospitals and within hospitals. This is further illustrated by the adjusted ICC for trauma center of 12.5%. This indicates a relatively poor correlation in time to surgical decompression between two similar patients treated at the same trauma center. When hospitals were grouped into quintiles based on time to surgical decompression adjusting for case-mix and hospital characteristics, the mean difference in surgical timing was over 24 h longer for those treated at centers in the 5th quintile compared to the 1st quintile. This again reinforces the notion that case-mix differences do not adequately account for the between-hospital variability in time to surgical intervention. Considering all these findings together, one may conclude that there exists significant variability between trauma centers with regard to timing of surgical intervention for acute thoracolumbar SCI, even after adjusting for individual case-mix. Moreover, there is also substantial unexplained variability at the patient-level within centers. This underscores the greater need for high-quality evidence and clinical practice guidelines in this domain.

This study is strengthened by the use of high-quality, audited data from over 200 trauma centers across North America, improving generalizability of the findings. Further, the large sample size of nearly 4000 patients permitted adjustmemt for a very large number of variables in our regression modelling. Nonetheless, this study does have some notable limitations. First, the hospitals included in this analysis were level I and II trauma centers; therefore, the results may not be generalizable to all institutions where patients with acute SCI are treated. Second, despite accounting for a number of important factors, certain variables that would be expected to influence the timing of decompressive surgery for acute thoracoliumbar SCI are not captured within the TQIP database and the analysis could not be adjusted for these variables; for example, grading of spinal cord injury severity beyond complete/incomplete and fracture classification^40–42^. Nonetheless, we were able to account for several relevant patient-, injury-, and hospital-related factors, which represent important sources of heterogeneity in this patient population. Thirdly, we did not include transport time and time spent in a referring hospital. Nearly one-third of our cohort was transferred from another institution prior to definitive surgical intervention. If the transfer time comprised a substantial proportion of time to surgery, then this unobserved time may bias the results. However, notably transfer status had no signficant effect on surgical timing in our study. Moreover, it has been previously shown that in patients with traumatic SCI, most pre-surgical time occurs between arrival at the treating hospital and arrival in the operating room^43,44^. Thus, it seems the probability of early surgery is not largely influenced by transfer and transport time and more likely relates to policies and practices at the treating center.

## Conclusion

The timing of surgical decompression for acute thoracolumbar SCI is influenced to a small degree by patient- and injury-related factors, including: age; race; severity of concomitant chest, abdominal, and lower extremity injury; and GCS and hemodynamic status on ED arrival. Nonetheless, there is substantial variability, both within and between hospitals, in the timeliness of surgery that is unexplained by patient-, injury-, and hospital-related factors. This variability underscores the need for greater and higher quality evidence and guidelines addressing the management of acute thoracolumbar SCI.

## Supplementary Information

Supplementary Information.

## Data Availability

The data used for this study is available from the American College of Surgeons (ACS) Trauma Quality Improvement Program (TQIP), and may be accessed upon request to the ACS.
