# Supplementary material for: Variability in time to surgery for patients with acute thoracolumbar spinal cord injuries
Source: Sci Rep. 2021 Jun 25;11:13312. doi: 10.1038/s41598-021-92310-z (PMC8233434; doi:10.1038/s41598-021-92310-z)
Supplement: Supplementary file 1 — Supplementary Information. [file 41598_2021_92310_MOESM1_ESM.docx]

**Variability in time to surgery for patients with acute thoracolumbar spinal cord injuries**

Supplementary Material

8,885 Patients with acute thoracolumbar SCI

8,849 With survivable injuries (AIS < 6)

36 Excluded

4,996 Treated with surgical decompression

1,654 Excluded

4,801 With data on timing of surgical decompression

195 Excluded

6,650 With blunt injury mechanism

2,199 Excluded

4,305 Underwent decompression within 5 days

496 Excluded

3,948 Treated at a center with *n* > 5 meeting criteria

357 Excluded

**Supplementary Figure 1: Flowchart of patient eligibility and enrollment**

**Supplementary Figure 2: Cumulative percentage of patients undergoing surgical decompression for acute thoracolumbar SCI as a function of time from ED**

| **Supplementary Table 1: AIS codes** | |
| --- | --- |
| **Cervical Spine** | |
| Incomplete cord syndrome^a^ | 640210.4, 640212.4, 640214.4, 640216.4, 640218.4; 640242.5, 640244.5, 640246.5, 640248.5, 640250.5 |
| Complete cord syndrome^b^ | 640220.5, 640221.5, 640222.5, 640224.5, 640226.5, 640228.5, 640229.6, 640230.6, 640232.6, 640234.6, 640236.6; 640260.5, 640261.5, 640262.5, 640264.5, 640266.5, 640268.5, 640269.6, 640270.6, 640272.6, 640274.6, 640276.6 |
| **Thoracolumbar Spine** | |
| Incomplete cord syndrome^a^ | 640410.4, 640412.4, 640414.4, 640416.4, 640418.4, 640442.5, 640444.5, 640446.5, 640448.5, 640450.5, 640610.4, 640612.4, 640614.4, 640616.4, 640618.4, 640642.5, 640644.5, 640646.5, 640648.5, 640650.5 |
| Complete cord syndrome^b^ | 640420.5, 640422.5, 640424.5, 640426.5, 640428.5, 640460.5, 640462.5, 640464.5, 640466.5, 640468.5, 640620.5, 640622.5, 640624.5, 640626.5, 640628.5, 640660.5, 640662.5, 640664.5, 640666.5, 640668.5 |
| ^a^Preservation of some sensation or motor function; includes anterior cord, central cord, lateral cord (Brown-Sequard) syndromes  ^b^Quadriplegia or paraplegia with no sensation | |

| **Supplementary Table 2: ICD-9-CM codes for surgical intervention** | | |
| --- | --- | --- |
| **Procedure** | **ICD-9-PCS Codes** | **ICD-10-PCS Codes** |
| Decompression | 03.0x, 03.53 | 0RN0, 0RN1, 0RN3, 0RN4, 0RN5, 0RN6, 0RN9, 0RNA, 0RNB, 0RT3, 0RT4, 0RT5, 0RT9, 0RTB, 0RP0, 0RP1, 0RP3, 0RP4, 0RP5, 0RP6, 0RP9, 0RPA, 0RPB, 0RB0, 0RB1, 0RB3, 0RB4, 0RB5, 0RB6, 0RB9, 0RBA, 0RBB, 0RW0, 0RW1, 0RW3, 0RW4, 0RW5, 0RW6, 0RW9, 0RWA, 0RWB, 0RQ0, 0RQ1, 0RQ3, 0RQ4, 0R5Q, 0RQ6, 0RQ9, 0RQA, 0RQB, 0SN0, 0SN2, 0SN3, 0SN4, 0ST2, 0ST4, 0SB0, 0SB2, 0SB3, 0SB4, 0SP0, 0SP2, 0SP3, 0SP4, 0SW0, 0SW2, 0SW3, 0SW4  0SQ0, 0SQ2, 0SQ3, 0SQ4, 00W, 00NX, 00NY |
| Fusion | 81.0x, 81.3x, 81.6x | ORG0, ORG1, ORG2, ORG4, ORG6, ORG7, ORG8, ORGA, 0RH0, 0RH1, 0RH3, 0RH4, 0RH5, 0RH6, 0RH9, 0RHA, 0RHB, OSG0, OSG1, OSG3, XRG0, XRG1, XRG2, XRG4, XRG6, XRG7, XRG8, XRGA, XRGB, XRGC, XRGD, 0SH0, 0SH2, 0SH3, 0SH4 |
| Traction | 93.41  93.42 | 2W62X0Z  2W65X0Z |

| **Supplementary Table 3: Modified* Charlson Comorbidity Index** | |
| --- | --- |
| **Comorbidity** | **Score** |
| Age (yrs) |  |
| < 50 | 0 |
| 50-59 | 1 |
| 60-69 | 2 |
| 70-79 | 3 |
| ≥ 80 | 4 |
| Myocardial infarction |  |
| No | 0 |
| Yes | 1 |
| Congestive heart failure |  |
| No | 0 |
| Yes | 1 |
| Peripheral vascular disease |  |
| No | 0 |
| Yes | 1 |
| Cerebrovascular accident |  |
| No | 0 |
| Yes | 1 |
| Chronic obstructive pulmonary disease |  |
| No | 0 |
| Yes | 1 |
| Diabetes |  |
| No | 0 |
| Yes | 1 |
| Chronic kidney disease |  |
| No | 0 |
| Yes | 2 |
| Metastatic cancer |  |
| No | 0 |
| Yes | 6 |
| *Modified from Charlson ME, Pompei P, Ales KL, MacKenzie CR. A new method of classifying prognostic comorbidity in longitudinal studies: development and validation. *J Chronic Dis*. 1987;40(5):373-83. doi:10.1016/0021-9681(87)90171-8 | |
